# Experimental and Numerical Investigation on Fe-SMA Strengthening of U-Rib Butt-Welded Joints with Porosity Defects

**DOI:** 10.3390/ma19132902

**Published:** 2026-07-06

**Authors:** Haoran Sui, Yi Liu, Yan Yao, Xu Zhou, Xue Bai, Jianxin Peng

**Affiliations:** 1 School of Civil and Environmental Engineering, Changsha University of Science and Technology, Changsha 410114, China; 2 College of Civil and Construction Engineering, Hunan Institute of Technology, Hengyang 421002, China; 3 Guangzhou Branch of China Road and Bridge Corporation, Guangzhou 510000, China

**Keywords:** orthotropic steel bridge deck, U-rib butt-welded joint, porosity defect, fatigue crack propagation, Fe-SMA strengthening

## Abstract

**Highlights:**

- Porosity defects influence stress and fatigue behavior of U-rib joints.
- Fe-SMA strengthening modifies stress response and improves fatigue life.
- A numerical model is developed and validated for welded joint behavior.
- The results provide insight into fatigue behavior of welded joints with defects.
- Fe-SMA strengthening shows potential for repair of damaged welded joints.
- Parametric analysis helps understand influence of activation temperature and defect size.

**Abstract:**

To investigate the influence of porosity defects and the strengthening effect of bonded iron-based shape memory alloy (Fe-SMA) plates, fatigue tests were conducted on defect-free, porosity-containing, and Fe-SMA-strengthened U-rib butt-welded specimens. A numerical model considering porosity defects and the bonded Fe-SMA plate was also established and validated against the experimental results. The results show that porosity defects significantly increased the local stress level near the crack. Under a load of 60 kN, the stress at the section 2 mm from the crack edge increased from 98 MPa to 139.5 MPa. Meanwhile, the fatigue life decreased from 260 × 10^4^ cycles to 127 × 10^4^ cycles. After Fe-SMA strengthening, the stress decreased to 75.59 MPa, and the fatigue life increased to 326 × 10^4^ cycles, which was 2.57 times that of the unreinforced defective specimen. The Fe-SMA plate did not change the fatigue crack propagation path but effectively slowed crack growth through local stiffness enhancement and activation-induced pre-compressive stress. Parametric analysis further showed that, among the investigated numerical cases, an activation temperature of 200 °C produced the largest predicted strengthening effect. Increasing the pore diameter from 0.5 mm to 2.0 mm reduced the reinforcement effect from 69.45% to 52.98%, and increasing the crack length from 10 mm to 50 mm reduced it from 65.41% to 35.53%. These results indicate that bonded Fe-SMA plates can effectively improve the fatigue performance of U-rib butt-welded joints with porosity defects, especially when applied before excessive crack growth occurs.

## 1. Introduction

Orthotropic steel bridge decks (OSBDs) have been widely used in modern long-span steel bridges because of their light self-weight, high load-carrying capacity, and favorable structural efficiency [1,2,3,4]. However, OSBDs contain numerous welded details, and under traffic loading, the geometric discontinuities of welds may lead to severe stress concentrations [5,6,7]. Among the typical fatigue-prone details, U-rib butt-welded joints are particularly vulnerable in OSBDs, because these joints are often completed by field welding, where welding quality is more difficult to control [8,9,10]. Once fatigue cracks initiate at such joints, they may propagate along the weld region, reduce the local stiffness of the U-rib, and further cause stress redistribution in the OSBD, thereby compromising the serviceability and safety of the bridge [11,12]. In practical engineering, welding defects are often difficult to avoid in butt-welded joints, and porosity is one of the common defect types. Such defects may aggravate local stress concentration and accelerate crack initiation and propagation. Therefore, it is necessary to clarify the fatigue behavior of U-rib butt-welded joints containing porosity defects and to develop an efficient strengthening method for extending their fatigue life.

In recent years, considerable efforts have been devoted to the fatigue behavior of defect-containing welded details in OSBDs. Fan et al. [13] conducted fatigue tests on rib-to-deck welded joints in steel bridges and showed that initial crack-like manufacturing defects at the weld root can strongly affect fatigue failure behavior. Luo et al. [14] combined experimental and numerical approaches to study the fatigue cracking behavior associated with weld defects in OSBDs, demonstrating that weld defects can significantly affect crack initiation and fatigue resistance. Lu et al. [15] analyzed the coupled propagation behavior of multiple fatigue cracks in welded steel bridge joints and showed that crack interaction may substantially accelerate fatigue deterioration. For defect characterization and fatigue assessment of welded steel details, Ma et al. [16] assessed steel welds with isolated surface porosity defects at the weld toe and found that pore geometry and location significantly affected fatigue strength and crack evolution. Xu et al. [17] investigated the formation and suppression mechanisms of welding porosity, revealing the influence of welding process conditions on pore formation. Jiang et al. [18] evaluated the fatigue life of deck-to-U-rib welds considering welding residual stress, while Qiang et al. [19] analyzed the stress intensity factors of surface cracks in OSBDs considering welding residual stresses. For U-rib butt-welded joints specifically, Zhang et al. [20] carried out fatigue tests and evaluation of U-rib butt welds in OSBDs, while Jiang et al. [21] experimentally investigated the fatigue cracking characteristics of U-rib butt welds in OSBDs. These studies have substantially improved the understanding of defect-sensitive fatigue behavior in steel bridge welded joints. However, the existing research on defect-containing OSBD welds has mainly focused on rib-to-deck welds, rib-to-diaphragm welds, and other fillet-welded details, whereas systematic investigations on U-rib butt-welded joints with porosity defects remain relatively limited. In particular, the effects of porosity defects on local stress response, crack propagation behavior, and fatigue life of U-rib butt-welded joints have not yet been sufficiently clarified. This may lead to inaccurate fatigue life evaluation and unreliable safety assessment of in-service steel bridges.

To improve the fatigue performance of OSBDs, various strengthening and repair techniques have also been proposed. Jiang et al. [22] investigated the strengthening of U-rib butt-welded connections using externally bonded CFRP strips, showing that the equivalent fatigue life could be significantly improved after repair. Wang et al. [23] proposed a fatigue strengthening solution for metallic structures using bonded prestressed Fe-SMA repairs and demonstrated that complete crack arrest could be achieved under favorable conditions. Lv et al. [24] further proposed a proactive strengthening technique for cracked U-rib butt-welded joints using adhesively bonded Fe-SMA plates, showing that the mode-I stress intensity factor at the crack tip was markedly reduced and the equivalent fatigue life could be extended by up to 4.09 times. Izadi et al. [25] also applied Fe-SMA plates to fatigue-cracked riveted steel bridge connections and confirmed the effectiveness of prestressed Fe-SMA strengthening in reducing fatigue-related damage. Shakir et al. [26] investigated the fatigue crack repair of rib-to-rib butt-welded connections in OSBDs using CFRP and steel plates, and developed a fracture-mechanics-based numerical framework to simulate crack propagation and evaluate repair effectiveness. These studies have confirmed the potential of Fe-SMA for fatigue strengthening of steel bridge details, owing to its combined effects of local stiffness enhancement and active prestress introduction. However, the existing studies mainly concern fatigue-cracked details without explicitly considering the influence of porosity defects in the weld region. Whether the strengthening effect observed in cracked details without weld porosity can be directly extended to U-rib butt-welded joints with porosity defects remains unclear. In addition, the effects of key strengthening parameters, such as activation temperature, pore diameter, and crack length at strengthening, have not been systematically investigated for this specific welded detail. Therefore, further investigation is needed to clarify the strengthening effect of bonded Fe-SMA plates on fatigue cracks in U-rib butt-welded joints with porosity defects.

In this study, the effects of porosity defects on fatigue crack propagation in U-rib butt-welded joints and the strengthening effectiveness of bonded Fe-SMA plates are investigated through experiments and numerical analysis. Fatigue tests were first conducted on defect-free, porosity-containing, and Fe-SMA-strengthened specimens to evaluate the influences of porosity defects and strengthening on local stress response, crack propagation behavior, and fatigue life. A finite element model considering both porosity defects and bonded Fe-SMA plates was then established and validated against the experimental results. Finally, based on the validated model, a parametric study was further performed to examine the effects of activation temperature, pore diameter, and crack length at strengthening on the strengthening performance. The results are expected to provide a useful basis for the fatigue assessment and repair design of U-rib butt-welded joints with porosity defects in OSBDs.

## 2. Experimental Design

### 2.1. Specimen Design

**Figure 1:**
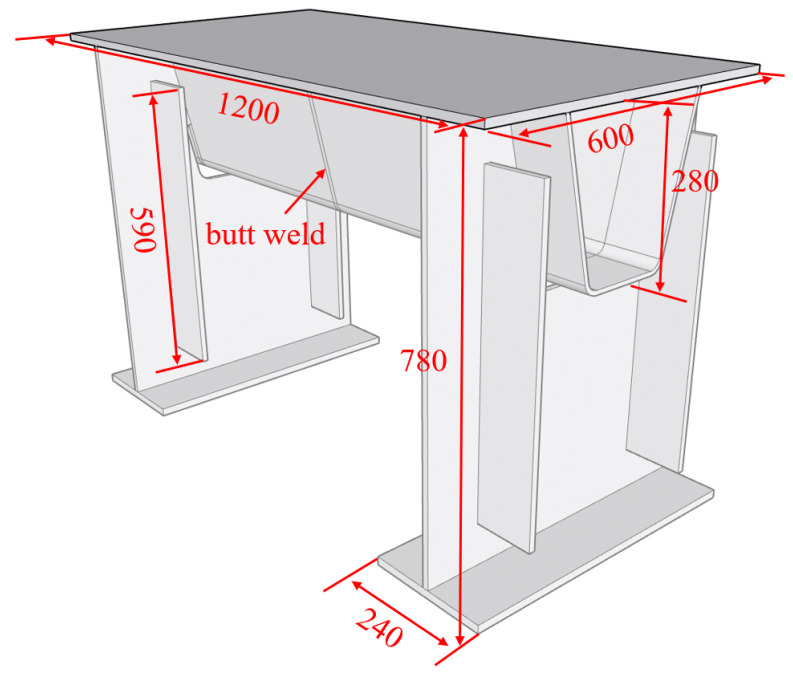
Geometry of the U-rib butt-welded specimen (unit: mm).

The U-rib butt-welded joint specimen was designed as a full-scale model based on a standard segment of a long-span steel box girder bridge, as shown in Figure 1. The specimen had a length of 1.2 m and a width of 0.6 m. The top plate thickness was 14 mm, and the U-rib had dimensions of 1200 mm × 280 mm × 8 mm in length, height, and thickness, respectively. A 12 mm thick diaphragm was arranged in the specimen, and two stiffeners were added to improve local stability. All specimens were fabricated using Q345C steel plates supplied by Hunan Decheng Steel Structure Co., Ltd. (Changsha, China). The U-rib-to-U-rib connections were fabricated as double-Y-groove butt welds. The weld width was 9 mm, and the root face height was 2 mm. The U-rib-to-deck connection was welded using CHW-50C6 flux-cored wire, corresponding to ER50-6 welding wire. All specimens were manufactured under the same welding procedure to ensure consistency. The Fe-SMA plates were supplied by Suzhou Haichuan Rare Metal Products Co., Ltd. (Suzhou, China). The Fe-SMA reinforcement plates used for the strengthened specimen had dimensions of 100 mm × 20 mm × 3 mm. The main material properties are listed in Table 1.

**Figure 2:**
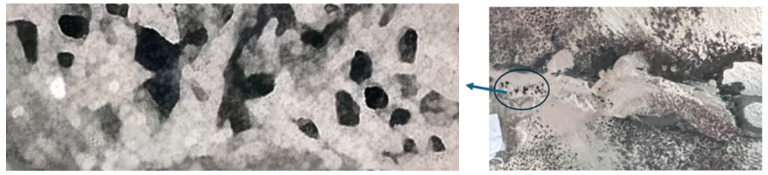
Photograph of porosity defects in the U-rib butt weld.

To investigate the effects of porosity defects and Fe-SMA strengthening, three groups of specimens were prepared, as listed in Table 2. Specimen A was defect-free and unreinforced. Specimen B contained porosity defects but was not reinforced. Specimen C also contained porosity defects and was strengthened using bonded Fe-SMA plates. For the defective specimens, porosity defects were introduced at the boundary of the U-rib bottom plate, as shown in Figure 2. For specimens B and C, porosity defects were intentionally introduced in the U-rib butt weld during manual welding by adding a small amount of water into the weld pool. This method was used to simulate welding-induced gas porosity and generated seven pores with a nominal target diameter of approximately 1 mm. However, because pore formation was affected by local welding conditions and could not be precisely controlled, the actual pores were irregular in shape and non-uniform in size and distribution. Therefore, these defects were regarded as representative irregular welding defects. For specimen C, the Fe-SMA plate was bonded after crack initiation to evaluate its strengthening effect.

### 2.2. Reinforcement Location and Procedure

**Figure 3:**
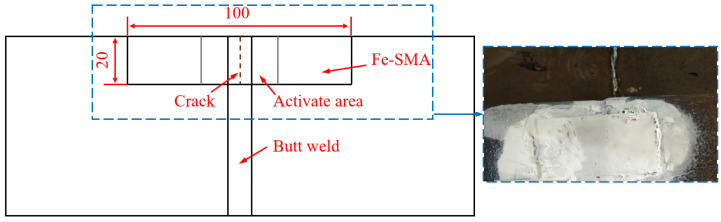
Configuration of bonded Fe-SMA strengthening (unit: mm).

When fatigue cracking occurred at the bottom of the U-rib, specimen C was strengthened using a bonded Fe-SMA plate. As shown in Figure 3, the Fe-SMA plate had dimensions of 100 mm × 20 mm × 3 mm and was bonded across the crack region at the bottom of the U-rib. The strip was arranged perpendicular to the butt weld so that the cracked zone could be effectively covered.

Before bonding, the steel surface in the strengthening region was ground to remove the oxide layer. Structural adhesive was then applied, and the Fe-SMA plate was pressed onto the steel plate to form a uniform bonding layer. Excess adhesive and entrapped air were removed during bonding. After curing under pressure for at least 4 days, the Fe-SMA plate was activated by local heating in the designated activation area shown in Figure 3. The activation temperature was set to 200 °C. Heating activation introduced prestress into the strengthened specimen, and the resulting pre-compressive stresses at the corresponding measurement sections of specimen C are summarized in Table 3.

### 2.3. Loading Scheme

**Figure 4:**
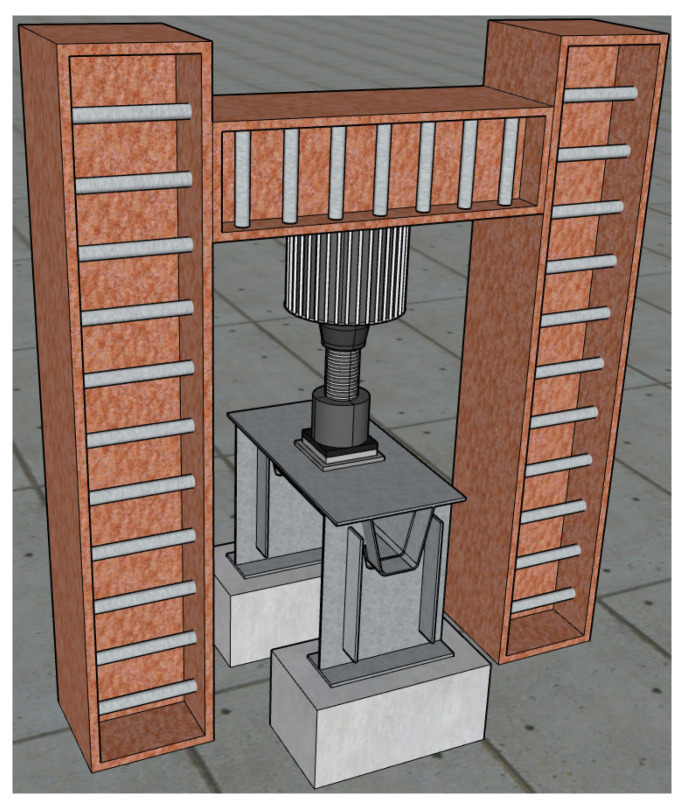
Fatigue loading setup.

Fatigue testing was conducted using a PMS-500 series electro-hydraulic servo pulsating fatigue testing machine (Jinan, China). A single-point loading scheme was adopted. The actuator load was applied at the center of the butt weld and transferred to the specimen through a loading pad assembly composed of steel and rubber plates, resulting in an effective loading area of 300 × 300 mm^2^, as shown in Figure 4. The rubber pads were used to simulate the compliant tire-deck contact condition and to ensure uniform load transfer, reducing local stress concentration. The specimen was supported under simply supported boundary conditions. The strain measurement device employed was the static strain TDS540 (Tokyo, Japan). Before fatigue loading, static loading was carried out to obtain the initial stress response of the specimen. The fatigue loading was applied in a cyclic mode with an upper limit of 60 kN and a lower limit of 20 kN, corresponding to a load amplitude of 40 kN, and the loading frequency was set to 5 Hz. The fatigue load was determined based on the measured ultimate load-bearing capacity of the specimen (approximately 340 kN). The applied maximum load of 60 kN corresponds to 17.6% of the ultimate capacity, ensuring that the applied fatigue loading remained primarily within the elastic response range of the specimen. Crack initiation was defined as the first visually detectable crack observed on the outer surface of the U-rib butt weld under cyclic loading. The crack length was measured on the external surface of the specimen using a steel ruler with a minimum scale of 1 mm. Due to accessibility limitations, measurements were conducted only on the outer side of the U-rib. During the fatigue test, static loading was performed after every 5 × 10^4^ cycles to monitor the stress response and crack evolution. The crack was first allowed to propagate under cyclic loading. When the crack length reached approximately 20 mm (measured on the external surface), the fatigue test was paused, and the Fe-SMA plate was bonded and activated. Fatigue loading was then resumed until the crack propagated through the entire U-rib bottom plate. The crack propagation path on the outer surface was marked using a white developer and a marker pen during the post-reinforcement fatigue stage, and the crack length was recorded at every 5 × 10^4^ cycles. The fatigue test was terminated when the crack propagated through the full width of the U-rib bottom plate. No specific ASTM or ISO standard was strictly followed, since the present study focuses on a structural-scale welded joint with in situ crack observation and strengthening intervention.

### 2.4. Measurement Point Layout

**Figure 5:**
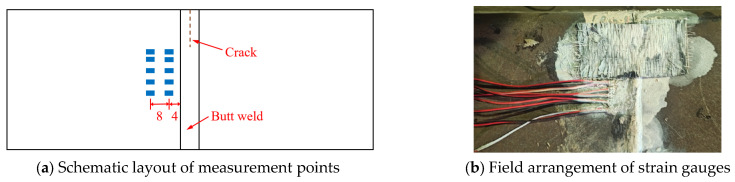
Layout of hot-spot stress measurement points.

The hot-spot stress method was adopted in this study. As shown in Figure 5, five measurement sections were arranged along the crack propagation direction on each specimen, and two strain gauges were installed at each section, resulting in a total of ten strain gauges per specimen. The five measurement sections were located at distances of 2, 5, 10, 15, and 20 mm from the crack edge, respectively. At each section, the two strain gauges were placed at distances of 0.5*t* and 1.5*t* from the weld toe, corresponding to 4 mm and 12 mm, respectively, where t = 8 mm. The schematic layout and field arrangement of the strain gauges are shown in Figure 5a and Figure 5b, respectively. The hot-spot stress was determined using the two-point linear extrapolation method, and the corresponding formula is given as follows:

(1) $$\sigma_{hs}=1.5\sigma_{0.5t}-0.5\sigma_{1.5t}$$

where $\sigma_{hs}$ is the extrapolated hot-spot stress at the weld toe, $\sigma_{0.5t}$ is the measured stress at a point 0.5*t* away from the weld, $\sigma_{1.5t}$ is the measured stress at a point 1.5*t* away from the weld, and *t* is the plate thickness (8 mm). The hot-spot stresses at the measurement sections located 2, 5, 10, 15, and 20 mm from the crack edge are denoted as $\sigma_{2}$, $\sigma_{5}$, $\sigma_{10}$, $\sigma_{15}$, and $\sigma_{20}$, respectively.

## 3. Experimental Results

### 3.1. Static Loading Results

**Figure 6:**
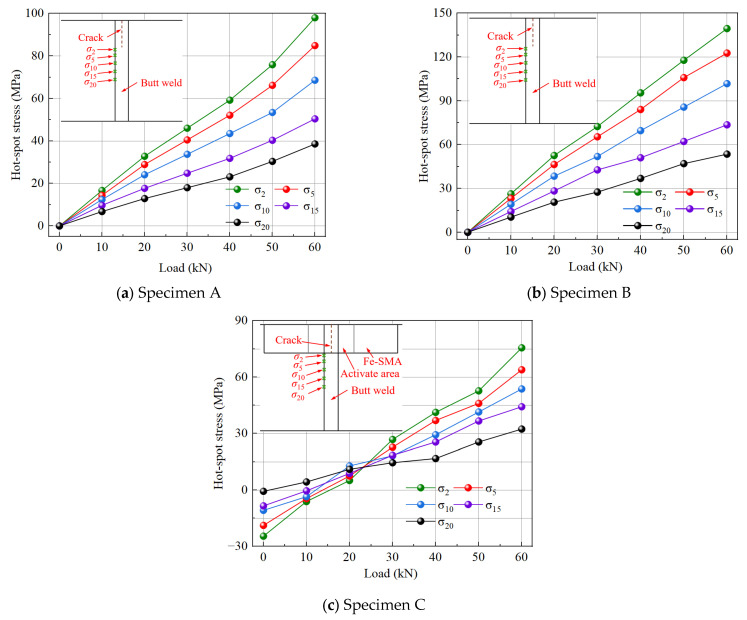
Hot-spot stress responses of specimens under different static loads.

The stress responses of the three specimens under static loading are presented in Figure 6. For all specimens, the stress at each measurement point increased approximately linearly with the applied load, indicating that the specimens remained in a stable mechanical state within the tested load range. For Specimen C, negative stress values were observed at zero external load because the activation of the bonded Fe-SMA plate introduced an initial pre-compressive stress. In addition, for all three specimens, the stress level increased as the measurement section approached the crack, showing a clear stress concentration effect in the crack vicinity.

To quantify the influence of porosity defects and Fe-SMA strengthening, the stress at the section located 2 mm from the crack edge, denoted as $\sigma_{2}$, under a static load of 60 kN was selected for comparison, as summarized in Table 4. The $\sigma_{2}$ value increased from 98 MPa in specimen A to 139.5 MPa in specimen B, corresponding to an increase of 42.3 MPa. This indicates that the presence of porosity defects intensified the local stress concentration near the crack. The pores reduced the effective load-carrying area of the weld and made the surrounding material more susceptible to localized deformation under loading. After strengthening, the $\sigma_{2}$ value decreased to 75.59 MPa in specimen C, which was 63.91 MPa lower than that of specimen B. This result demonstrates that the bonded Fe-SMA plate effectively reduced the local tensile stress at the cracked region. The reduction can be attributed to two main factors: the bonded Fe-SMA plate increased the local stiffness of the cracked section, and its thermal activation introduced pre-compressive stress, which further counteracted the tensile stress concentration near the crack.

### 3.2. Fatigue Failure Modes

**Figure 7:**
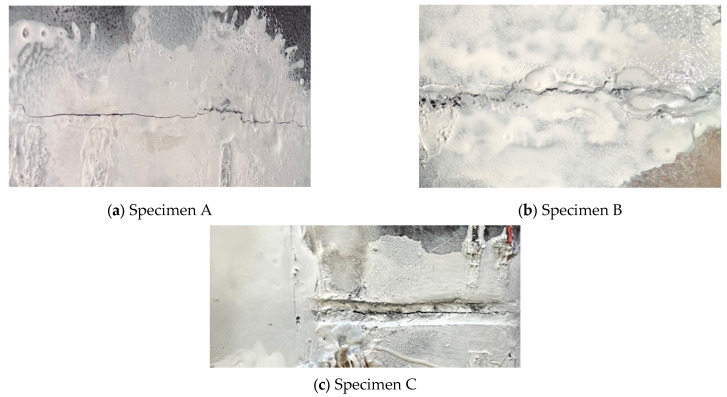
Fatigue failure modes of specimens.

The fatigue failure modes of the specimens are shown in Figure 7a–c. For all specimens, fatigue cracks appeared at the bottom of the U-rib and propagated along the longitudinal direction of the U-rib bottom plate. With continued cyclic loading, the cracks gradually extended toward the opposite side and finally penetrated the U-rib bottom. This indicates that the specimens exhibited a similar final fatigue failure mode.

By comparing Figure 7a,b, it can be seen that the crack in the defect-free specimen initiated at the U-rib bottom, whereas the crack in the specimen with porosity defects initiated near the porosity defects. This indicates that the pores acted as local stress concentration sources under cyclic loading, making the defective region more prone to fatigue crack initiation. The presence of porosity defects reduced the effective load-bearing area of the weld and intensified the local stress concentration, thereby promoting crack initiation and subsequent propagation. By comparing Figure 7b,c, it can be observed that the Fe-SMA-strengthened specimen showed a similar crack propagation path to the unreinforced defective specimen, and the crack still propagated along the U-rib bottom. Therefore, the bonded Fe-SMA plate did not change the main fatigue failure path of the specimen.

Although the final crack path was not changed by Fe-SMA strengthening, no obvious debonding of the Fe-SMA plate was observed during the fatigue test, indicating that the bonded reinforcement remained effective. The Fe-SMA plate provided a bridging effect across the cracked region and introduced pre-compressive stress after activation, which helped reduce crack opening and suppress crack propagation.

### 3.3. Crack Propagation and Fatigue Life

**Figure 8:**
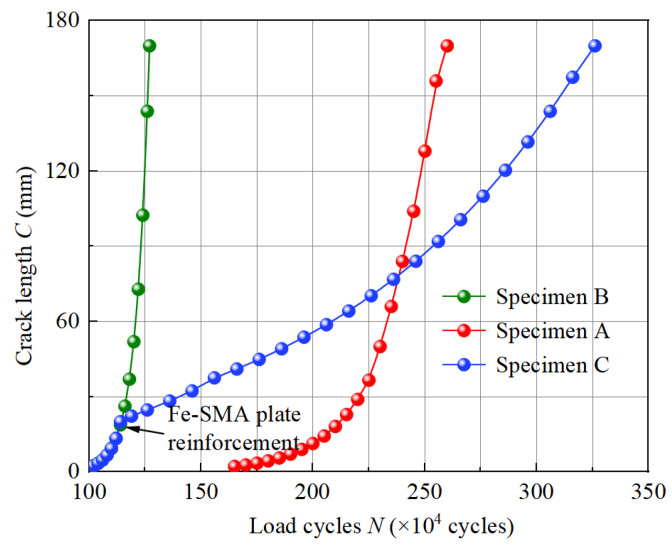
Crack propagation curves of specimens.

The crack propagation curves of the specimens are shown in Figure 8. For all specimens, the crack length increased with the number of fatigue cycles, but the growth behavior differed among the three specimens. Compared with specimen A, specimen B showed earlier crack initiation and a steeper crack growth curve, indicating that porosity defects accelerated fatigue damage. This is because the pores reduced the effective load-bearing area of the weld and induced local stress concentration under cyclic loading, thereby promoting crack initiation. Compared with specimen B, the crack growth curve of specimen C became much flatter after Fe-SMA strengthening, although the crack continued to propagate along the U-rib bottom. This indicates that the bonded Fe-SMA plate effectively delayed crack propagation. The strengthening effect can be attributed to the local stiffness enhancement provided by the bonded plate and the pre-compressive stress introduced by thermal activation, which partially counteracted the tensile stress near the crack and reduced the crack opening tendency.

The fatigue life of each specimen is summarized in Table 5. Specimen A reached a fatigue life of $260\times 10^{4}$ cycles, whereas specimen B failed at $127\times 10^{4}$ cycles; thus, the fatigue life of the defect-free specimen was 2.05 times that of the specimen with porosity defects. This confirms that porosity defects significantly reduced the fatigue resistance of the U-rib butt-welded joint. For specimen C, the total fatigue life reached $326\times 10^{4}$ cycles, which was 2.57 times that of specimen B. This result demonstrates that Fe-SMA strengthening effectively suppressed crack growth and extended the fatigue life of the porosity-containing butt-welded joint. The observed increase in fatigue life and the retardation of crack propagation are consistent with previous studies on Fe-SMA strengthening of fatigue-cracked U-rib butt-welded joints reported in Ref. [24].

## 4. Numerical Analysis

### 4.1. Finite Element Model

**Figure 9:**
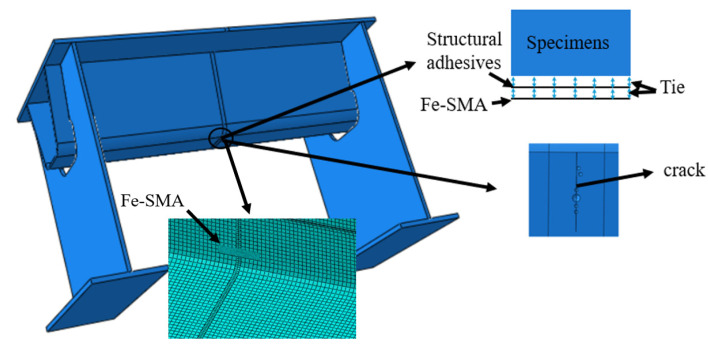
Finite element model of the Fe-SMA-strengthened U-rib butt-welded joint.

A three-dimensional finite element model of the U-rib butt-welded joint was established using ABAQUS 2023, as shown in Figure 9. The geometric dimensions of the model were consistent with those of the experimental specimen. The model consisted of the steel specimen, the epoxy adhesive layer, and the Fe-SMA plate. The steel specimen and Fe-SMA plate were discretized using C3D8R elements, while the epoxy adhesive layer was discretized using COH3D8 elements. The thicknesses of the Fe-SMA plate and adhesive layer were 3 mm and 2 mm, respectively. The constitutive model of Q345C steel was adopted from a previous study by the authors [27], while the material parameters of the Fe-SMA plate and epoxy adhesive were defined according to Table 1. A global mesh size of 10 mm was adopted for the model. The weld region was locally refined to 2 mm [28], and the region around the crack tip was further refined, with a fine mesh size of 0.5 mm adopted near the crack tip.

For the specimens with porosity defects, since the prefabricated porosity defect could only be approximately controlled, they were idealized as hemispherical voids in the finite element model. The nominal target pore diameter of 1.0 mm used during specimen fabrication was adopted as the average equivalent void diameter in the model. As shown in the local schematic of Figure 9, the voids were arranged along the crack path in the weld region according to the approximate defect location. The hemispherical voids were then subtracted from the model using Boolean operations. For the strengthened specimen, the Fe-SMA plate was bonded to the U-rib bottom through the adhesive layer according to the reinforcement configuration used in the test. The interfaces between the steel specimen, adhesive layer, and Fe-SMA plate were connected using tie constraints. An initial crack with a length of 20 mm and a depth of 1 mm was introduced at the U-rib bottom near the butt weld to represent the crack state before reinforcement.

The numerical analysis was performed in two steps. First, the prestress induced by Fe-SMA activation was simulated by applying an equivalent temperature field to the activation area of the Fe-SMA plate. Then, the external load was applied to reproduce the experimental loading condition. The loading position and area were consistent with those used in the test, and the boundary conditions were defined according to the experimental support arrangement.

### 4.2. Model Validation

**Figure 10:**
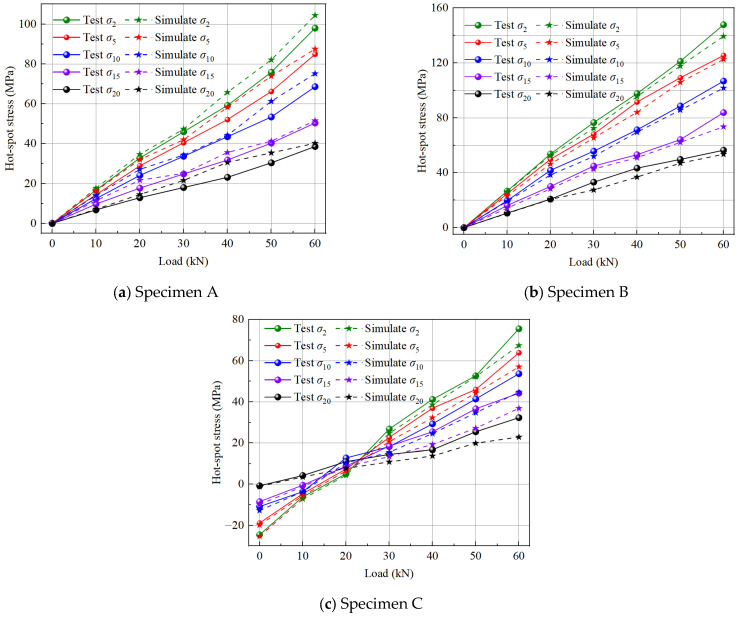
Comparison between tests and finite element hot-spot stresses.

The finite element model was validated using the hot-spot stress $\sigma_{2}$ under static loading, as shown in Figure 10. The finite element results show good agreement with the experimental results for all three specimens. The stress values increase almost linearly with the applied load, and the model captures the higher stress level of the specimen with porosity defects and the negative initial stress of the Fe-SMA-strengthened specimen caused by activation. The discrepancies between the experimental and finite element results may be attributed to the simplification of boundary conditions, loading contact, pore morphology, weld geometry, adhesive thickness, and interfacial behavior in the numerical model. Overall, the relative error of the key stress $\sigma_{2}$ was within approximately 15%, indicating that the model can reasonably reflect the stress response of the specimens and can be used for the subsequent parametric analysis.

### 4.3. Parametric Analysis of Reinforcement Effect

#### 4.3.1. Effects of Activation Temperature and Activation Zone Length

**Figure 11:**
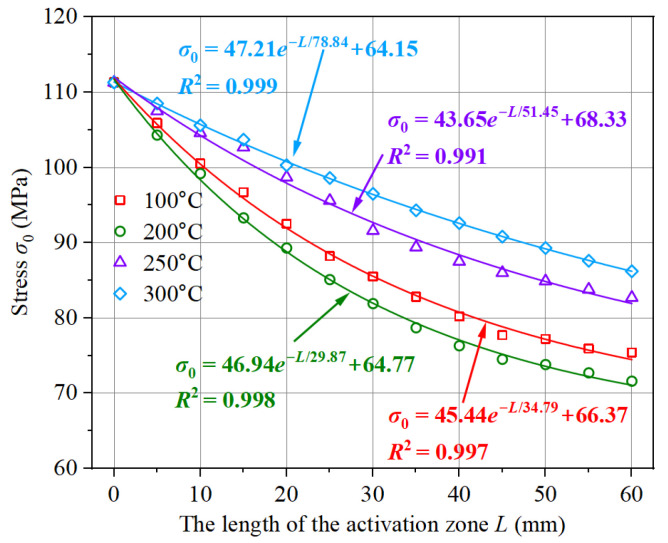
Effects of activation temperature and activation zone length on stress $\sigma_{0}$.

A parametric analysis was conducted under a static load of 60 kN to investigate the effects of activation temperature and activation zone length on the Fe-SMA strengthening effect. The stress $\sigma_{0}$ at the critical location was selected as the evaluation index. As shown in Figure 11, $\sigma_{0}$ decreased continuously with the increase in activation zone length under all activation temperatures, indicating that enlarging the activation zone can effectively reduce the stress level near the crack. When the activation zone length increased from 0 mm to 60 mm, $\sigma_{0}$ decreased from 111.3 MPa to 75.4 MPa, 71.6 MPa, 82.7 MPa, and 86.2 MPa at activation temperatures of 150 °C, 200 °C, 250 °C, and 300 °C, respectively. The corresponding stress reductions were 35.9 MPa, 39.7 MPa, 28.6 MPa, and 25.1 MPa. Among them, the 200 °C case produced the largest stress reduction, indicating the best strengthening effect.

The reduction in $\sigma_{0}$ was more significant at the early stage of activation zone extension, while the decreasing trend gradually slowed as L increased. For example, at 200 °C, $\sigma_{0}$ decreased from 111.3 MPa to 81.9 MPa when L increased from 0 mm to 30 mm, whereas it further decreased to 71.6 MPa when L increased from 30 mm to 60 mm. This indicates that the strengthening effect tends to become saturated after the activation zone reaches a certain length. In terms of activation temperature, the stress level at L=60 mm under 200 °C was 94.96%, 86.58%, and 83.06% of those under 150 °C, 250 °C, and 300 °C, respectively. Therefore, excessive activation temperature did not further improve the reinforcement effect. Based on these results, 200 °C was adopted as the preferred activation temperature in this study, and the activation temperature is recommended to be controlled within 150–200 °C.

#### 4.3.2. Effect of Pore Diameter

**Figure 12:**
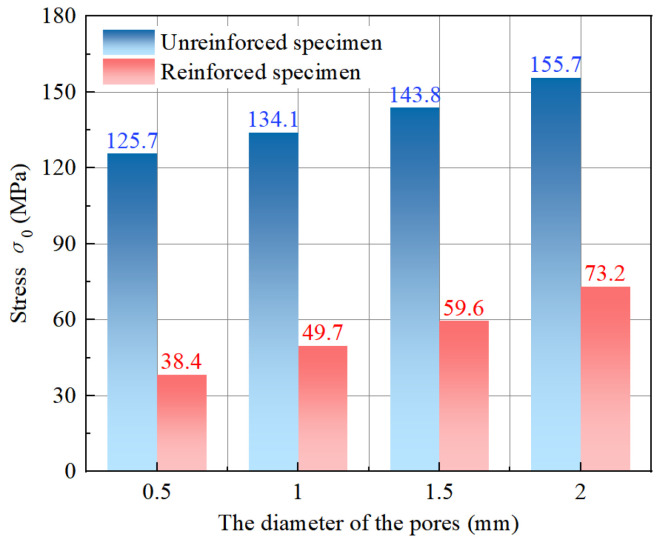
Effect of pore diameter on stress $\sigma_{0}$ before and after Fe-SMA strengthening.

The effect of pore diameter on the Fe-SMA strengthening performance was analyzed under a static load of 60 kN, with an activation temperature of 200 °C and an activation area of 30×20 mm^2^. In the finite element model, the average equivalent void diameter was set to 1.0 mm. To further investigate the influence of pore size on the strengthening effect, pore diameters of 0.5, 1.0, 1.5, and 2.0 mm were considered in the parametric analysis. This range covers both smaller and larger pores relative to the baseline equivalent diameter of 1.0 mm. The pore defects were located on the central axis of the butt weld and in the middle of the crack. The stress $\sigma_{0}$ at the critical location was selected as the evaluation index. As shown in Figure 12, $\sigma_{0}$ increased with the pore diameter for both the unreinforced and reinforced specimens. For the unreinforced specimen, $\sigma_{0}$ increased from 125.7 MPa to 155.7 MPa as the pore diameter increased from 0.5 mm to 2.0 mm. For the reinforced specimen, $\sigma_{0}$ increased from 38.4 MPa to 73.2 MPa. This indicates that larger pore defects caused more severe local stress concentration, because the increase in pore size further reduced the effective load-bearing area of the weld region and intensified the geometric discontinuity near the crack.

The reinforcement effects corresponding to different pore diameters are summarized in Table 6. When the pore diameter increased from 0.5 mm to 2.0 mm, the reinforcement effect decreased from 69.45% to 52.98%. This result indicates that the bonded Fe-SMA plate remained effective in reducing the local stress level, but its strengthening efficiency gradually decreased as the pore diameter increased. The reason is that larger pores introduced stronger initial stress concentration, making it more difficult for the stiffness enhancement and activation-induced pre-compressive stress provided by the Fe-SMA plate to fully compensate for the defect-induced stress amplification.

#### 4.3.3. Effect of Crack Length on Reinforcement Efficiency

**Figure 13:**
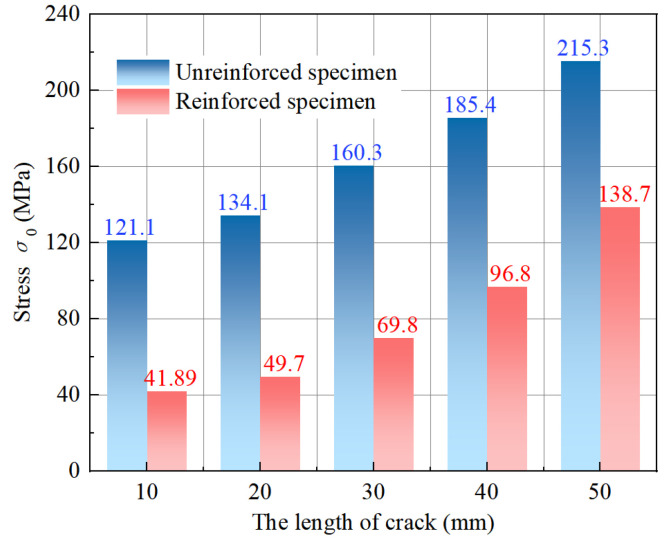
Effect of crack length on stress $\sigma_{0}$ before and after Fe-SMA strengthening.

The effect of crack length on Fe-SMA reinforcement efficiency was analyzed under a static load of 60 kN, with an activation temperature of 200 °C and an activation area of 30×20 mm^2^. As shown in Figure 13, the stress $\sigma_{0}$ increased with the crack length for both the unreinforced and reinforced specimens. For the unreinforced specimen, $\sigma_{0}$ increased from 121.1 MPa to 215.3 MPa as the crack length increased from 10 mm to 50 mm. After Fe-SMA strengthening, $\sigma_{0}$ was reduced to 41.89 MPa, 49.7 MPa, 69.8 MPa, 96.8 MPa, and 138.7 MPa for crack lengths of 10, 20, 30, 40, and 50 mm, respectively. This indicates that the bonded Fe-SMA plate can effectively reduce the local stress level for different crack lengths.

The reinforcement effects are summarized in Table 7. When the crack length increased from 10 mm to 50 mm, the reinforcement effect decreased from 65.41% to 35.53%. In particular, the reinforcement effect only slightly decreased from 65.41% to 62.93% when the crack length increased from 10 mm to 20 mm, indicating that the strengthening effect remained relatively stable at the early crack-growth stage. However, when the crack length further increased to 30, 40, and 50 mm, the reinforcement effect decreased to 56.46%, 47.70%, and 35.53%, respectively. This shows that excessive crack growth weakens the effectiveness of Fe-SMA strengthening. The reason is that a longer crack leads to more severe stress concentration and a larger crack-opening tendency, making it more difficult for the local stiffness enhancement and activation-induced pre-compressive stress provided by the Fe-SMA plate to counteract the tensile stress near the crack tip. Therefore, Fe-SMA strengthening should be applied before excessive crack propagation occurs. Within the scope of this study, strengthening at a crack length of approximately 20 mm remained effective and may be considered as a reference timing for early intervention.

## 5. Conclusions

In this study, fatigue tests were conducted on U-rib butt-welded joints to investigate the influence of porosity defects and the strengthening effect of bonded Fe-SMA plates. Three types of specimens, namely defect-free unreinforced, porosity-containing unreinforced, and porosity-containing Fe-SMA-strengthened specimens, were designed and tested under static and fatigue loading. A numerical model considering porosity defects, the adhesive layer, and the bonded Fe-SMA plate was then established and validated using the experimental stress results. Based on the validated model, the effects of activation temperature, pore diameter, and crack length on the reinforcement efficiency were further analyzed. The main conclusions are as follows:

(1) Porosity defects significantly increased the local stress level and accelerated fatigue damage in the U-rib butt-welded joint. Under a static load of 60 kN, the stress at the section 2 mm from the crack edge increased from 98 MPa to 139.5 MPa due to the presence of porosity defects. Correspondingly, the fatigue life decreased from 260 × 10^4^ cycles to 127 × 10^4^ cycles, indicating that porosity defects weakened the local load-bearing capacity and promoted fatigue crack initiation and propagation. This highlights the detrimental effect of welding porosity on fatigue performance.
(2) Bonded Fe-SMA strengthening effectively reduced the stress level and improved the fatigue life of the defective welded joint. After strengthening, the stress at the same section decreased from 139.5 MPa to 75.59 MPa under 60 kN, and the fatigue life increased to 326 × 10^4^ cycles, which was 2.57 times that of the unreinforced defective specimen. Although Fe-SMA strengthening did not change the crack propagation path, it reduced crack opening through local stiffness enhancement and activation-induced pre-compressive stress. This demonstrates the effectiveness of Fe-SMA for fatigue strengthening of welded joints with defects.
(3) The established numerical model reasonably captured the stress response of defect-free, defective, and Fe-SMA-strengthened specimens, with a relative error of approximately 15% in key stress response predictions. The parametric analysis indicated that, under the numerical conditions, 200 °C produced the largest predicted strengthening effect among the investigated activation temperatures. Meanwhile, larger pore diameters and longer cracks reduced the predicted strengthening efficiency. The reinforcement effect decreased from 69.45% to 52.98% as pore diameter increased from 0.5 mm to 2.0 mm, and from 65.41% to 35.53% as crack length increased from 10 mm to 50 mm.

It should also be noted that only three representative specimens were tested in this study, corresponding to the defect-free, porosity-defective, and Fe-SMA strengthened cases. Although the results clearly demonstrate the fatigue performance trend and strengthening effectiveness of Fe-SMA, the limited number of specimens may not fully capture the inherent scatter in fatigue behavior of welded joints. Therefore, the presented results should be regarded as preliminary, and further experimental investigations with larger sample sizes are recommended in future work.

## Figures and Tables

**Table 1:** Material properties used in this study.

Name	Density (g·cm^−3^)	Young’s Modulus (GPa)	Yield Strength (MPa)	Poisson’s Ratio
Q345C	7.85	205	345	0.3
Fe-SMA	7.2	172	494	0.3
Structural adhesive	1.85	4.863	/	0.3

**Table 2:** Specimen groups and key parameters.

No.	Presence of Porosity Defects	Fe-SMA Reinforcement Applied
Specimen A	No defects	No reinforcement
Specimen B	With defects	No reinforcement
Specimen C	With defects	Reinforced

**Table 3:** Pre-compressive stresses induced by Fe-SMA activation in specimen C.

Activation Temperature (°C)	Pre-Compressive Stress (MPa)				
	$\sigma_{2}$	$\sigma_{5}$	$\sigma_{10}$	$\sigma_{15}$	$\sigma_{20}$
200	24.56	18.83	10.83	8.43	0.71

**Table 4:** Hot-spot stress $\sigma_{2}$ of each specimen under 60 kN static load.

No.	Specimen A	Specimen B	Specimen C
$\sigma_{2}$ (MPa)	98	139.5	75.59

**Table 5:** Fatigue life comparison of specimens.

No.	Specimen Description	*N_f_* (×10^4^ Cycles)	Life Ratio
Specimen A	Defect-free, unreinforced	260	2.05
Specimen B	Porosity-containing, unreinforced	127	1.00
Specimen C	Porosity-containing, Fe-SMA strengthened	326	2.57

Note: *N_f_* denotes the fatigue life. The life ratio is calculated relative to specimen B.

**Table 6:** Reinforcement effects under different pore diameters.

Pore Diameter (mm)	0.5	1.0	1.5	2.0
Reinforcement effect η (%)	69.45	62.93	58.55	52.98

Note: $\eta =(\sigma_{0,u}-\sigma_{0,r})/\sigma_{0,u}\times 100\%$, where $\sigma_{0,u}$ and $\sigma_{0,r}$ are the stresses of the unreinforced and reinforced specimens, respectively.

**Table 7:** Reinforcement effects under different crack lengths.

Crack Length (mm)	10	20	30	40	50
Reinforcement effect η (%)	65.41	62.93	56.46	47.70	35.53

## Data Availability

The original contributions presented in this study are included in the article. Further inquiries can be directed to the corresponding authors.

## Abbreviations

The following abbreviations are used in this manuscript:

Fe-SMA	Iron-based shape memory alloy
OSBD	Orthotropic steel bridge deck
η	Reinforcement effect
L	The length of crack
